# Disability adjusted life years associated with COVID-19 in Denmark in the first year of the pandemic

**DOI:** 10.1186/s12889-022-13694-9

**Published:** 2022-07-09

**Authors:** Sara M. Pires, Hernan G. Redondo, Laura Espenhain, Lea S. Jakobsen, Rebecca Legarth, Marianna Meaidi, Anders Koch, Siri Tribler, Tomas Martin-Bertelsen, Steen Ethelberg

**Affiliations:** 1 Risk Benefit Research Group, National Food Institute, Technical University of Denmark, Lyngby, Denmark; 2 Department of Infectious Disease Epidemiology and Prevention, Statens Serum Institut, Copenhagen S, Denmark; 3 Data Integration and Analysis, Division of Infection Preparedness, Statens Serum Institut, Copenhagen S, Denmark; 4 Department of Public Health, Global Health Section, University of Copenhagen, Copenhagen, Denmark

**Keywords:** Burden of disease, DALY, YLD, YLL, Population health, Denmark, European burden of disease network, Coronavirus, COVID-19

## Abstract

**Background:**

Burden of disease studies measure the public health impact of a disease in a society. The aim of this study was to quantify the direct burden of COVID-19 in the first 12 months of the epidemic in Denmark.

**Methods:**

We collected national surveillance data on positive individuals for SARS-CoV-2 with RT-PCR, hospitalization data, and COVID-19 mortality reported in the period between 26^th^ of February, 2020 to 25^th^ of February, 2021. We calculated disability adjusted life years (DALYs) based on the European Burden of Disease Network consensus COVID-19 model, which considers mild, severe, critical health states, and premature death. We conducted sensitivity analyses for two different death-registration scenarios, within 30 and 60 days after first positive test, respectively.

**Results:**

We estimated that of the 211,823 individuals who tested positive to SARS-CoV-2 by RT-PCR in the one-year period, 124,163 (59%; 95% uncertainty interval (UI) 112,782–133,857) had at least mild symptoms of disease. The total estimated disease burden was 30,180 DALYs (95% UI 30,126; 30,242), corresponding to 520 DALYs/100,000. The disease burden was higher in the age groups above 70 years of age, particularly in men. Years of life lost (YLL) contributed with more than 99% of total DALYs. The results of the scenario analysis showed that defining COVID-19-related fatalities as deaths registered up to 30 days after the first positive test led to a lower YLL estimate than when using a 60-days window.

**Conclusion:**

COVID-19 led to a substantial public health impact in Denmark in the first full year of the epidemic. Our estimates suggest that it was the the sixth most frequent cause of YLL in Denmark in 2020. This impact will be higher when including the post-acute consequences of COVID-19 and indirect health outcomes.

**Supplementary Information:**

The online version contains supplementary material available at 10.1186/s12889-022-13694-9.

## Background

Worldwide, the first reported outbreak caused by severe acute respiratory syndrome coronavirus 2 (SARS‑CoV‑2) was identified in December 2019 in Wuhan, China. On March 11 (2020), Coronavirus disease 2019 (COVID‑19) was categorized as a pandemic by the World Health Organization [1]. In Denmark, the first case was notified February 26, 2020. Over the following year, Denmark registered over 200,000 cases and 2,600 deaths, and implemented a range of public health measures to limit spread of infections. These included an emergency law extending the powers of the public health authorities and law enforcement agencies, allowing the government to implement measures including restricting access to public institutions and public transport; closing Danish borders to all non-resident foreigners and foreigners not working in Denmark; enforcing a lockdown in the spring of 2020, reinforced in December 2020; implementing restrictive measures such as closing of schools, administrative establishments and public places; and implementing a widespread, free of charge, testing strategy launched by the Statens Serum Institut (SSI) [2–4].

Since the beginning of the pandemic, countries have been running efforts to measure the health impact of the disease in their populations, including monitoring and daily publication of numbers of suspected and confirmed COVID-19 cases, hospitalizations, hospitalizations requiring intensive care, and deaths. In Denmark, the SSI is responsible for surveillance of COVID-19 in humans, including case counts, admissions and deaths. In addition, national surveys of prevalence of antibodies to SARS-CoV-2 were carried out [5]. These metrics have been useful to monitor the evolution of the epidemic over time, the effects of measures to reduce transmission of infection, and guide options for introducing or lifting restrictions at different stages.

For assessing the health significance and severity of a disease on the society, tools that account for the overall health impact are needed [6]. Burden of disease studies use measures of mortality, morbidity and disability caused by given diseases and are useful to compare the public health impact of a disease across population groups, across diseases in a country, or of the same disease across countries.

The aim of this study was to quantify the direct burden of COVID-19 in the first 12 months of the epidemic in Denmark.

## Methods

To estimate the burden of disease of COVID-19, we obtained information on all individuals that tested positive for SARS-CoV-2 with RT-PCR in the period between the 26^th^ of February, 2020 to the 25^th^ of February, 2021 from The Danish Microbiological Database (MiBa), which contains all microbiological test results from the national TestCenter Denmark and all clinical microbiological departments in Denmark [7, 8]. Information included age, sex and date of performance of the test. Data on dates of admission and discharge from hospitals for stays longer than 12 h, including intensive care units (ICU), were obtained by linkage to the National Patient Registry [9]. We defined an admission as ‘COVID-19-related’ if the patient had confirmed SARS-CoV-2 and the first positive test was performed in the time window from 14 days prior to admission date and until the date of discharge. If the first positive test was performed later than two days after the admission date, the date of testing was used as the ‘COVID-19-related’-admission date. We defined intensive care treatment as ‘COVID-19-related’ if the intensive care was provided during a COVID-19-related admission. Information about vital status within 60 days was obtained from the Civil Registry and The National Cause of Death Register [10]. We defined death as ‘COVID-19-related’ if it occurred within 30 days from the first positive SARS-CoV-2 test. Deaths up to 60 days after testing positive have also been recorded, under the assumption that they may be associated with infection with SARS-CoV-2. To test the impact using a 60-days window in the overall burden of disease estimate, we also carried out a scenario analysis using a 60-days window.

### Disability-Adjusted Life Year (DALY)

The Disability-Adjusted Life Year (DALY) is a health gap metric, measuring the healthy life years lost due to disease [6]. DALYs are calculated by adding the number of years of life lost due to premature mortality (YLLs) and the number of years lived with disability, adjusted for severity (YLDs):

1 $$DALY=YLL+YLD$$

**Fig. 1:**
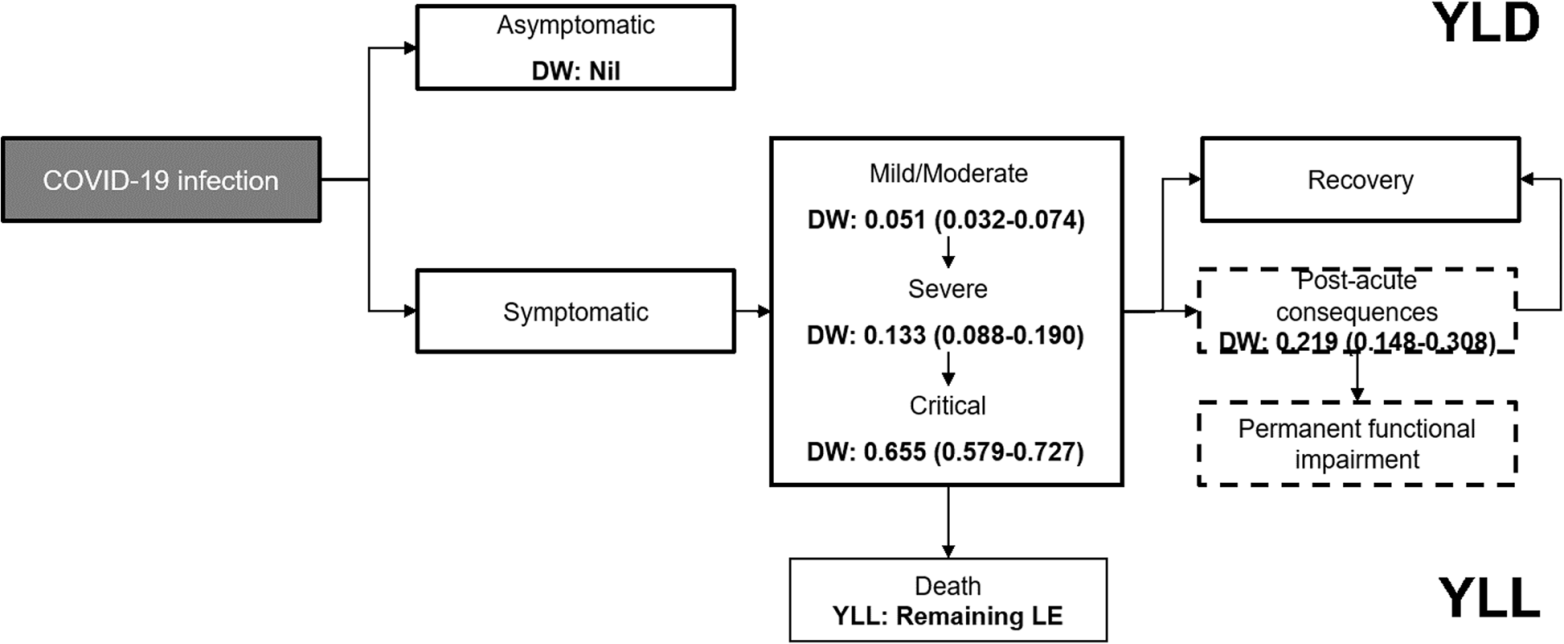
Disease model for COVID-19 (adapted from [11]). The dashed boxes describe post-acute health outcomes of COVID-19, which were not included in the study

The DALY accounts for all health states experienced upon infection and is calculated on the basis of a disease model (Fig. 1). We calculated DALYs for the whole study period, segregating the population by sex and 10-year age groups.

### Years lived with disability (YLD)

To define morbidity due to COVID-19, we established the direct health states that are experienced by individuals with COVID-19 in an incidence-based model as defined by Wyper et al. [11]. In an incidence-based approach, all health outcomes, including those in future years, are assigned to the initial event. This approach reflects the future burden of disease resulting from current events.

To estimate the number of symptomatic cases among all registered PCR-positive individuals, we obtained estimates of the proportion of symptomatic SARS-CoV-2 infections from the Danish National Seroprevalence Survey of SARS-CoV-2 infection (DSS) [5]. In this survey, a representative sample of the Danish population aged 12 year and older were invited to have a blood sample analysed for SARS-CoV-2 antibodies, and at the same time fill in an online questionnaire concerning symptoms of infection. We multiplied the number of cases in each age and sex group by a probability of a positive individual being symptomatic. This probability was defined based on the number of seropositive samples among surveyed individuals, and the number of these individuals who reported at least one relevant symptom [5] (Table 1). We assumed the same probability of a positive case presenting symptoms for all individuals across age groups and sex.

**Table 1:** COVID-19 disability weights by health states and corresponding symptoms

Name	Description	Data input/ calculation	Duration (days)	Disability weight (95% uncertainty interval)	Source
Asymptomatic	Has infection but experiences no symptoms	PCR-positive cases—Estimated symptomatic cases	NA	Nil	*Surveillance*^ab^
Mild to moderate	Has a fever and aches, and feels weak, which causes some difficulty with daily activities	PCR-positive cases^d^ Probability of a sero-positive case having symptoms (Beta(216,369))	10^c^	0.051^d^ (0.032–0.074)	*Surveillance*^a^ *Survey of sero-prevelance* + *symptoms*^b^
Severe	Has a high fever and pain, and feels very weak, which causes great difficulty with daily activities	Number of hospitalised patients (non-intensive care)	Mean duration of hospital stays^e^	0.133^d^ (0.088–0.190)	Surveillance^a^
Critical	Intensive care unit admission	Number of hospitalised patients (intensive care)	Mean duration of ICU stays	0.655^d^ (0.579–0.727)	Surveillance^a^

^a^[12]

^b^[5]

^c^[13]

^d^DWs were defined as Pert distributions, informed by the mean estimate and 95% uncertainty interval

^e^If a patient was moved to ICU during her/his hospital stay, and then transferred again to the hospital, the days at ICU were excluded from hospital duration

To define the severity of each health state, we extracted disability weights (DWs) from the Global Burden of Disease Study [14] and from the European Disability Weight study [15] (Table 1). The DW of a given health outcome reflects the severity of the health outcome (i.e. reduction in health-related quality of life). For each health state defined for symptomatic individuals, we combined data on the incidence from surveillance data, duration and disability from literature, and calculated YLD by summing the product of the number of cases, duration (in years) and disability weight, across all health states:

2 $$YLD= \sum_{h=1}^{l}{YLD}_{H}= \sum_{h=1}^{l}{Number of cases}_{h}\times {duration}_{h}\times {disability weight}_{h}$$

      where *h* = health state and *l* = number of health states.

We classified time spent with symptoms as either “mild to moderate”, “severe” and “critical” assuming that all infected persons with symptoms were at least in the “mild to moderate” state. We assumed that all hospitalised cases had severe symptoms, and classified all patients receiving intensive care (ICU) as “critical”. The mean duration of “mild to moderate” symptoms was assumed to be 10 days [13]. We calculated the duration of severe and critical cases as the mean duration of hospitalizations and ICU admissions, respectively. The symptoms included in each of these three states, the data input proxies, and the corresponding disability weights are presented in Table 1. The names, descriptions and disability weights of the health outcomes “mild to moderate” and “severe” were based on those from the GBD 2019 study for infectious diseases of the lower respiratory tract [14]. The health outcome “critical” was defined by the European Disability Weight study [16].

### Years of life lost due to premature mortality (YLL)

YLL were estimated by multiplying the number of deaths (M) in each age group by the residual life expectancy (RLE) at the age of death:

3 $$YLL=M*RLE$$

We applied the age conditional life expectancy defined by the GBD 2019 reference life table, which assigns the same values to both males and females [17]. For deaths in patients aged 95 years or older we assigned the RLE of individuals aged 95.

### Uncertainty

To incorporate uncertainty in YLD estimates, we propagated the uncertainty in the probability of a SARS-CoV-2 infection being symptomatic, and of the disability weights. We applied Monte Carlo simulation with 10,000 iterations. The model was implemented in R version 4.0.3 [18], and the code is available in Additional file 1.

### Scenario analyses

Defining deaths that occurred within 30 days from the first positive SARS-CoV-2 test as ‘COVID-19-related’ may lead to an underestimation of associated mortality and YLL. To evaluate the impact of the assumption, we calculated mortality and YLL using deaths that occurred within 60 days from the first positive SARS-CoV-2 test, and compared YLL and DALY estimates.

## Results

We estimated that of the 211,823 individuals who tested positive to SARS-CoV-2 by RT-PCR in the one-year period, 124,163 (59%; 95% uncertainty interval (UI) 112,782–133,857), corresponding to 59% (95% UI 53 – 63%), had at least one disease symptom (Table 2). We estimated that 7% of symptomatic cases exhibited severe symptoms, 1% critical symptoms, and 2% had died within 30 days. In absolute numbers, the highest number of mild and moderate cases were registered in the age groups 20–29, 40–49 and 50–59 years of age. In contrast, the incidence of severe cases, critical cases and deaths increased with age, with the highest (absolute) number of deaths registered in the age group 80–89 (Table 2).

**Table 2:** Number of PCR-positive SARS-CoV-2 cases, estimated symptomatic cases, observed hospitalizations within 14 days from first positive SARS-CoV-2 test, intensive care unit cases, and observed deaths within 30 days from first positive SARS-CoV-2 test in Denmark, by age and sex (February 2020 to February 2021)

	**All cases**^**a**^		**Symptomatic cases**^**b**^		**Severe cases**^**a**^		**Critical cases**^**a**^		**Deaths**^**a**^	
**Age group**	**Female**	**Male**	**Female**	**Male**	**Female**	**Male**	**Female**	**Male**	**Female**	**Male**
0–9	6,839	7,396	4,009 [3,641; 4,322]	4,335 [3,938; 4,674.]	53	43	6	2	0	1
10–19	15,851	16,571	9,291 [8,439; 10,017]	9,714 [8,823; 10,472]	59	46	4	5	0	0
20–29	20,441	19,361	11,983 [10,883; 12,917]	11,349 [10,308; 12,235]	221	123	11	10	0	0
30–39	14,833	14,148	8,695 [7,898; 9,373]	8,293 [7,533; 8,941]	296	224	18	22	4	2
40–49	16,627	14,440	9,746 [8,853; 10,507]	8,464 [7,688; 9,125]	372	456	37	44	2	4
50–59	15,897	14,742	9,317 [8,464; 10,046]	8,641 [7,849; 9,316]	626	868	54	149	21	32
60–69	8,215	8,582	4,815 [4,373; 5,191]	5,030 [4,569; 5,423]	672	1,066	105	203	73	122
70–79	5,162	5,323	3,026 [2,748; 3,262]	3,120 [2,834; 3,364]	1,086	1,575	137	308	228	394
80–89	3,239	2,362	1,899 [1,725; 2,047]	1,384 [1,258; 1,493]	1,111	1,148	50	97	439	518
90 +	1,269	525	744 [676; 802]	308 [280; 332]	375	266	7	5	323	220
Total	108,373	103,450	63,525 [57,700; 68,484]	60,639 [55,080; 65,373]	4,871	5,815	429	845	1,090	1,293
Total (female + male)	211,823		124,164 [112,780; 133,857]		10,686		1274		2,383	

^a^Registered to Surveillance

^b^Estimated (mean and 95% Uncertainty Interval)

**Fig. 2:**
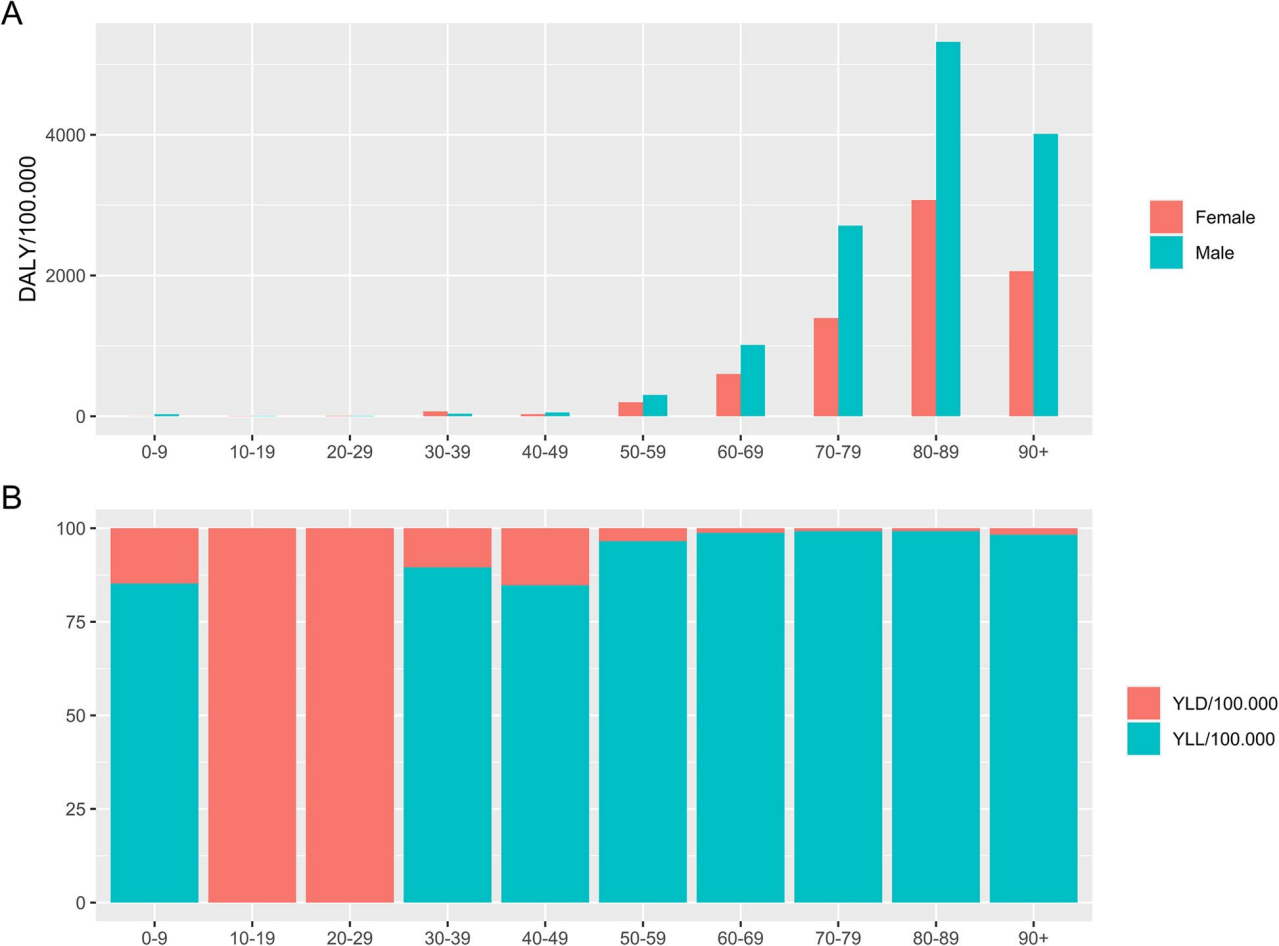
**A** Disability adjusted life years (DALY) per 100.000 due to COVID-19 in Denmark by age and sex (February 2020-February2021). **B** Relative contribution of YLD and YLL in females and males to the total DALY by age group

The total estimated disease burden was 30,181 DALYs due to COVID-19 (95% UI 30,126; 30,242) (Table 3), or 520 DALYs/100,000. The DALY was higher in the age groups above 70 years of age, particularly in men (Fig. 2). A total of 232 years of life were lost due to disability (YLD) in the one year period (Table 3), overall equal to around 1% of total DALY. Mild cases contributed overall with 35% of YLD, severe with 5%, and critical cases with 60% (Supplementary material, Additional file 2). The 2,383 deaths registered in the one-year period resulted in the loss of 29,689 years of life lost due to premature mortality (YLL). The total YLL was larger in males than in females, and larger in the age group 70–79 years old. Except in females aged 0–29 and males aged 10–19, YLL contributed with more than 99% of total DALY (Table 3).

**Table 3:** Total number of years of life lost due to disability (YLD), years of life lost due to premature mortality (YLL) and disability adjusted life years (DALY) due to COVID-19 in Denmark by age and sex (February 2020-February2021)

	**YLD**^a^		**YLL**^a^		**DALY**^a^	
**Age group**	**Female**	**Male**	**Female**	**Male**	**Female**	**Male**
0–9	7.2 [6.1; 8.9]	7.4 [6.3; 8.9]	0	84.5	7.2 [6.1; 8.9]	91.9 [90.8; 93.4]
10–19	14.4 [11.4; 18.3]	15.4 [12.3;18.1]	0	0	14.4 [11.4; 18.3]	15.4 [12.3; 18.1]
20–29	23 [20; 27]	20 [14.8; 23.2]	0	0	23 [19.7; 26.6]	20 [14.8; 23.2]
30–39	21 [18; 25]	17.4 [14.7; 21.2]	215.1	112.1	236.1 [232.7; 240.3]	129.5 [126.9; 133.3]
40–49	23 [20;27]	25.7 [22.6; 29.3]	91.1	180.3	114.2 [110.8; 118.2]	205.9 [202.9; 209.6]
50–59	31 [29; 34]	38.7 [34.9; 42.7]	761.3	1,179.2	792.7 [789.8; 795.6]	1,217.8 [1,214.1; 1,221.9]
60–69	27 [25; 30]	38.8 [35.4; 42.4]	2,000.7	3,276.2	2,028 [2,026; 2,030.5]	3,315 [3,311.6; 3,318.5]
70–79	37 [34;41]	51 [46.2; 56.2]	4128.8	7264.8	4,165.5 [4,163; 4,169.3]	7,315.8 [7,311; 7,321]
80–89	36 [34; 39]	36.5 [34.5; 38.7]	4076.4	5152.6	4,112.5 [4,110.1; 4,115.6]	5,189.1 [5,187.1; 5,191.3]
90 +	12 [11; 13]	8.4 [7.6; 9.3]	649.1	517	661.3 [660; 662]	525.5 [524.6; 526.3]
Total	233 [207; 263]	259 [229; 290]	11,922.5	17,766.7	12,155.1 [12,129.7; 12,185.6]	18,025.9 [17996; 18,056.7]
**Total (female + male)**	491.9 [436.7; 553.3]		29,689		30,181 [30,125.8; 30,242.3]	

^a^Estimated mean (95% Uncertainty Interval)

### Scenario analysis

The results of the scenario analysis showed that defining COVID-19-related fatalities as deaths registered within 60 days of the first positive test led to a higher YLL estimate than when using death within 30 days (Table 4). The 418 deaths’ difference lead to 6,885 more YLL. Differences were distributed across sex and age groups.

**Table 4:** Number of deaths and years of life lost (YLL) by sex and age group using registered deaths at 60 and at 30 days after first SARS-CoV-2 positive test

**Definition of death**	**30 days after SARS-CoV-2 positive test**				**60 days after SARS-CoV-2 positive test**			
	**Deaths**		**YLL**		**Deaths**		**YLL**	
**Age group**	**Female**	**Male**	**Female**	**Male**	**Female**	**Male**	**Female**	**Male**
0–9	0	1	0	84.5	0	1	0	84.5
10–19			0	0	0	0	0	0
20–29			0	0	0	2	0	123.1
30–39	4	2	215.1	112.1	5	3	267.5	163.6
40–49	2	4	91.1	180.3	5	9	227.2	409.3
50–59	21	32	761.3	1179.2	30	45	1094.5	1650.5
60–69	73	122	2000.7	3276.2	88	161	2419.2	4349.3
70–79	228	394	4128.8	7264.8	268	485	4872.2	8977.2
80–89	439	518	4076.4	5152.6	508	589	4721.2	5896.7
90 +	323	220	649.1	517	368	233	744.7	554.2
Total	1090	1292	11,922.5	17,766.7	1272	1528	14,346.4	22,208.2
**Total** **(females + males)**		2,382		29,689		2,800		36,555

## Discussion

COVID-19 has had an unquestionable impact on societies globally. In Denmark, we estimated that 30,181 life years were lost in the first full year of the epidemic. Over 99% of DALYs were associated with the premature death of nearly 2,400 individuals. On average, fatal cases lost 13 years due to premature death. Our estimates included only the direct immediate health impact of the pandemic. However, the impact of COVID-19 on health occurs through two main pathways: directly, as an infectious disease; and indirectly, as a risk factor for secondary negative outcomes, for example, through increases in mental health issues due to national lockdowns or delay to medical treatment (including surgery), follow-up and delay in diagnoses through restrictions to vital healthcare services [19]. The indirect impact is expected to be large and to expand beyond the termination of the epidemic. Furthermore, several patients report post-acute consequences of COVID-19 (“long COVID”) [20, 21]. Data on the proportion of patients that experience these symptoms, and on the duration and the disability associated with health outcomes, were not sufficient to include in our model. However, we acknowledge that not including them will have led to an underestimation of the burden of COVID-19.

### Data and model assumptions

To estimate the proportion of SARS-CoV-2 positive individuals that experienced symptoms, we used data from a national Danish seroprevalence survey. Using this estimate may have underestimated the proportion of symptomatic cases and consequently underestimated the YLD. The seroprevalence study measured the proportion of symptomatic SARS-CoV-2 *infections* as opposed to the proportion of symptomatic *diagnosed with a* SARS-CoV-2 infection. Though the test activity has been extremely high in Denmark throughout most of the epidemic, including testing of asymptomatic individuals, it is estimated that on average only one out of three SARS-CoV-2 infections was diagnosed and captured by the surveillance system within the study period [5]. This may be even more pronounced in the first two months of the epidemic, where test capacity was low and people with severe symptoms were prioritized for testing. The sample size in the seroprevalence study did not allow for stratifying the proportion of symptomatic SARS-CoV-2 infections on age or sex, which is why we assumed the same prevalence of symptomatic individuals across age and sex. Because it has been suggested that the proportion of non-symptomatic or mild symptomatic infected individuals is higher in younger individuals [22, 23], using the same proportion for all age groups may have led to an overestimation in younger age groups. On the other hand, as children below 12 years of age has not been recommended routine tests unless symptomatic, a higher proportion of diagnosed SARS-CoV-2 cases amongst younger children are expected to be symptomatic compared to the older children.

COVID-19 infection progresses from mild to severe and further to critical disease, as described in our disease model. However, in a high proportion of cases, there is a transition from more to less severe health states, with continuation of milder symptoms. In our model, we quantified the disability due to the transition from critical back to severe health states by adding the subsequent duration of hospital stay, after release of ICU. However, we did not account for further transition to mild symptoms of the disease. While it is possible that patients continue experiencing symptoms after being discharged from hospital and thus this assumption will lead to an underestimation of YLD, we expect this to have a minor impact on the total burden of disease, as the severity (reflected by the DW) is minor. Hospital admissions due to COVID-19 were registered as any patient admitted to the hospital for more than 12 h in the 14-days period after first positive SARS-CoV-2 test, or when the first positive test was during an ongoing hospitalisation, no matter the reason for admission.

We defined ‘COVID-19-related deaths’ as a death that occurred within 30 days from the first positive SARS-CoV-2 test of the patient. This definition operated on death of all causes, and was not limited to deaths with a detailed evaluation of the cause of death registered in The National Cause of Death Register. To determine the impact of choosing a more sensitive definition of COVID-19-related deaths, we conducted a scenario analysis using mortality within 60 days of positive SARS-CoV-2 test as input data. Not surprisingly, this analysis showed that using registration at 60 days after testing positive led to a higher (23%) COVID-19-related death count (i.e. additional 418 deaths and 6,865 YLL). It is recognised that COVID-19 may give rise to an extended course of illness, sometimes gradually worsening over weeks and resulting in deaths after a period of more than one month. At the same time however, increasing the time window of the definition will increase the risk of including incidents where death have occurred for other or partially other causes than the SARS-CoV-2 infection.

Independent of the 30- or 60-days windows, it is a limitation that we defined ‘COVID-19-related deaths’ as any death occurring within a time window from the positive first SARS-CoV-2 test, as stated in the National Cause of Death register. Thus, persons may die from other causes than COVID-19 within these time windows, or die from COVID-19 later than 60 days. In such cases, there is a risk that our figures could both overestimate and underestimate the true number of COVID-19 related deaths. However, we could not validate exact causes of death in COVID-19 positive persons as stated on death certificates, but more detailed analyses of causes of death in the future might improve the accuracy of the figures for covid-19 related deaths in Denmark.

Other studies estimated DALYs or YLL for COVID-19 in multiple countries in the initial stages of the pandemic, including Denmark [24–26]. While these studies retrieved data from publically available data resources (such as from the European Centre for Disease Prevention and Control (ECDC), the World Bank Group (WBG) and the World Health Organization (WHO) [24], or resources created to gather and analyse COVID-19 data internationally [26]), those data were provided by the same surveillance system we collected our data from (SSI). Thus, discrepancies in estimates are explained by differences in the time period covered, in the disease models and calculations performed, or level of detail of data used (for example the age of the patient at time of death. National burden of disease efforts have the advantages of having direct access to country-specific detailed and national expertise on surveillance systems and diseases, thus allowing for adopting approaches that fit within their country contexts [27].

### Burden of COVID-19 in Denmark in perspective

The Global Burden of Disease study (GBD) estimated that the leading causes of disease in Denmark in 2019 were ischemic heart disease (1,712 DALYs/100,000 population), low back pain (1,631 DALYs/100,000) and chronic obstructive pulmonary disorders (1,597 DALYs/100,000) [28]. When ranking diseases by YLL (I.e., accounting only for premature mortality) the ranking is led by ischemic heart disease (1,638 YLLs/100,000), lung cancer (1,457 YLL/100,000) and obstructive pulmonary disorders (1,069 YLLs/100,000). If there were no major changes in disease burden in 2020 (results not available at the time of writing of this article), our results indicate that COVID-19 was the sixth leading disease cause of YLL in Denmark in 2020. While estimates for other diseases for the year covered in the study were not available, other studies have found differences on premature mortality in countries globally. For example, Islam et al. [29] analyzed data for various countries, and estimated a reduction in total YLL in Denmark in 2020. Given our estimated YLL for COVID-19 during the first year of the pandemic, such results suggest that other causes of premature mortality have been reduced.

Several countries have estimated the burden of COVID-19 in the same or similar time periods. In Europe, several groups have made efforts to harmonize methodologies and adopt comparable approaches to estimate DALYs, which we have followed and adapted [11]. At the time of writing of this article (October 2021), the Netherlands [13], Scotland [30], Germany [31] and Malta [32] have published DALY estimates. All estimates are consistent in the contribution of YLL to total burden (between approximately 99% in the Netherlands and Germany to 95% in Malta). The highest DALY/100,000 was estimated in Scotland (1,770–1,980 DALYs/100,000), and the lowest in Germany (368 DALY/100,000). The progress of the epidemic, the population structures, data availability and data assumptions, and model choices are reflected in differences in estimates of these countries. In general, DALY differences between countries in 2020 reflect how hard each country was hit by the epidemic, and the age distribution of deaths in each country. Relative to a number of other European countries, the Danish seroepidemiological studies suggest that Denmark was only affected mildly by the 2020 epidemic [5].The vaccine program was initiated in Denmark on December 27^th^, 2020. By the end of the period of this study (end of February, 2021), 7% of the population had received their first dose of COVID-19 vaccinations. While the impact of COVID-19 vaccination in the Danish population is therefore limited in the study period, we expect that it will be visible in the following year. As 75% of the Danish population in the relevant age groups has been fully vaccinated by October 30, 2021, it is important to measure the disease burden in 2021 using the same methods and metrics.

## Conclusion

COVID-19 has caused a substantial disease burden in Denmark in the first year. Our estimates excluded the burden associated with post-acute symptoms of COVID-19, as well as the burden caused with indirect health effects of COVID-19 pandemic and implemented disease mitigation strategies. Still, we found that COVID-19 was the 6^th^ leading cause of premature mortality in the country in 2020. When applied to data from subsequent time periods, we expect our model will be able to show the effect of population-wide vaccination.

## Supplementary Information

**Additional file 1.**

**Additional file 2: Table S1.** Years of life lost due to disability (YLD) caused by mild, severe and critical symptoms by age and sex of COVID-19 in Denmark, February 2020 to February 2021 (Mean and 95% Confidence Interval).

## Data Availability

De-identified participant-level data are available for access to members of the scientific and medical community for non-commercial use only. Applications should be submitted to Forskerservice at The Danish Health Data Authority (https:// sundhedsdatastyrelsen.dk/da/ forskerservice), where they will be reviewed on the basis of relevance and scientific merit.

## Abbreviations

COVID-19: Coronavirus Disease 2019
DALY: Disability adjusted life year
YLD: Years of life lost due to disability
YLL: Years of life lost due to premature mortality
ICU: Intensive care unit
GBD: Global Burden of Disease
RLE: Residual life expectancy

## References

[1] WHO. WHO Director-General’s opening remarks at the media briefing on COVID-19 - 11 March 2020. Press release. 2020. https://www.who.int/director-general/speeches/detail/who-director-general-s-opening-remarks-at-the-media-briefing-on-covid-19---11-march-2020. Accessed 4 Jun 2021.

[2] Hansen CH, Michlmayr D, Gubbels SM, Mølbak K, Ethelberg S (2021). Assessment of protection against reinfection with SARS-CoV-2 among 4 million PCR-tested individuals in Denmark in 2020: a population-level observational study. Lancet (London, England).

[3] Mens H, Koch A, Chaine M, Andersen Bengaard A (2021). The Hammer vs Mitigation-A comparative retrospective register study of the Swedish and Danish national responses to the COVID-19 pandemic in 2020. APMIS..

[4] Schønning K, Dessau RB, Jensen TG, Thorsen NM, Wiuff C, Nielsen L, Gubbels S, Denwood M, Thygesen UH, Christensen LE, Møller CH, Møller JK, Ellermann-Erik S, Voldstedlund M (2021). Electronic reporting of diagnostic laboratory test results from all healthcare sectors is a cornerstone of national preparedness and control of COVID-19 in Denmark. APMIS.

[5] Espenhain L, Tribler S, Sværke Jørgensen C, Holm Hansen C, Wolff Sönksen U, Ethelberg S (2021). Prevalence of SARS-CoV-2 antibodies in Denmark: nationwide, population-based seroepidemiological study. Eur J Epidemiol.

[6] Murray CJL (1994). Quantifying the burden of disease: The technical basis for disability-adjusted life years. Bull World Health Organ.

[7] Schønning K, Dessau RB, Jensen TG, Thorsen NM, Wiuff C, Nielsen L, Gubbels S, Denwood M, Thygesen UH, Christensen LE, Møller CH, Møller JK, Ellermann-Erik S, Voldstedlund M (2021). Electronic reporting of diagnostic laboratory test results from all healthcare sectors is a cornerstone of national preparedness and control of COVID-19 in Denmark. APMIS..

[8] Voldstedlund M, Haarh M, Mølbak K, the MiBa Board of Representatives C (2014). The Danish Microbiology Database (MiBa) 2010 to 2013. Eurosurveillance.

[9] Schmidt M, Schmidt SA, Sandegaard JL, Ehrenstein V, Pedersen L, Sørensen HT (2015). The Danish National Patient Registry: a review of content, data quality, and research potential. Clin Epidemiol.

[10] Helweg-Larsen K. The Danish Register of Causes of Death: 10.1177/1403494811399958. 2011;39:26–9. doi:10.1177/1403494811399958. 10.1177/1403494811399958 21775346

[11] Wyper GM, Assunção RM, Colzani E, Grant I, Haagsma JA, Lagerweij G, Von der Lippe E,McDonald SA, Pires SM, Porst M, Speybroeck N, Devleesschauwer B. Burden of disease methods: a guide to calculate COVID-19 disability-adjusted life years. Int J Public Health. 2021;66:619011. 10.3389/ijph.2021.619011. 10.3389/ijph.2021.619011 PMC8565264 34744580

[12] Pottegård A, Kristensen KB, Reilev M, Lund LC, Ernst MT, Hallas J, Thomsen RW, Christiansen CF, Sørensen HT, Johansen NB, Støvring H, Christensen S, Kragh Thomsen M, Brun NC (2020). Existing Data Sources in Clinical Epidemiology: The Danish COVID-19 Cohort. Clin Epidemiol.

[13] Lagerweij G, Schimmer B, Mooij S, Raven S, Schoffelen A, de Gier B (2020). Staat van Infectieziekten in Nederland, 2019. Bilthoven.

[14] Salomon JA, Haagsma JA, Davis A, de Noordhout CM, Polinder S, Havelaar AH (2015). Disability weights for the Global Burden of Disease 2013 study. Lancet Glob Heal.

[15] Maertens De Noordhout C, Devleesschauwer B, Salomon JA, Turner H, Cassini A, Colzani E (2017). Disability weights for infectious diseases in four European countries: comparison between countries and across respondent characteristics. Eur J Public Health..

[16] Haagsma JA, de Noordhout CM, Polinder S, Vos T, Havelaar AH, Cassini A (2015). Assessing disability weights based on the responses of 30,660 people from four European countries. Popul Health Metr.

[17] Global Burden of Disease Collaborative Network. Global Burden of Disease Study 2019 (GBD 2019) Reference Life Table. Seatle, United States; 2021.

[18] R Core Team (2021). R A language and environment for statistical computing.

[19] Douglas M, Katikireddi SV, Taulbut M, McKee M, McCartney G (2020). Mitigating the wider health effects of covid-19 pandemic response. BMJ..

[20] Lancet T (2020). Facing up to long COVID. Lancet.

[21] Nalbandian A, Sehgal K, Gupta A, Madhavan MV, McGroder C, Stevens JS (2021). Post-acute COVID-19 syndrome. Nat Med.

[22] Zimmermann P, Curtis N (2020). COVID-19 in Children, Pregnancy and Neonates: A Review of Epidemiologic and Clinical Features. Pediatr Infect Dis J.

[23] Bhopal SS, Bagaria J, Olabi B, Bhopal R (2021). Children and young people remain at low risk of COVID-19 mortality. Lancet Child Adolesc Heal.

[24] Gianino MM, Savatteri A, Politano G, Nurchis MC, Pascucci D, Damiani G (2021). Burden of COVID-19: Disability-Adjusted Life Years (DALYs) across 16 European countries. Eur Rev Med Pharmacol Sci.

[25] Pifarré I Arolas H, Acosta E, López-Casasnovas G, Lo A, Nicodemo C, Riffe T (2021). Years of life lost to COVID-19 in 81 countries. Sci Rep.

[26] Greg Williams, Angela Spencer, Tracey Farragher, Matthew Gittins AV. Years of life lost to COVID-19 in 20 countries — JOGH. J Glob Health. 2022;12. 10.7189/jogh.12.05007.

[27] Haneef R, Schmidt J, Gallay A, Devleesschauwer B, Grant I, Rommel A (2021). Recommendations to plan a national burden of disease study. Arch Public Heal.

[28] Network. GB of DC. Global Burden of Disease Study 2019 (GBD 2019) Results. 2020.

[29] Islam N, Jdanov DA, Shkolnikov VM, Khunti K, Kawachi I, White M (2021). Effects of covid-19 pandemic on life expectancy and premature mortality in 2020: time series analysis in 37 countries. BMJ.

[30] Wyper GMA, Fletcher E, Grant I, McCartney G, Fischbacher C, Harding O, et al. Measuring the direct population impact of COVID-19 in Scotland, 2020: estimating disability-adjusted life years (DALYs) during the first full calendar year. 10.31235/OSF.IO/EY36D.

[31] Rommel A, von der Lippe E, Plass D, Ziese T, Diercke M, der Heiden MA (2021). The COVID-19 Disease Burden in Germany in 2020. Dtsch Arztebl Int.

[32] Cuschieri, Sarah, Calleja, Neville, Devleesschauwer, Brecht, Wyper G. Estimating the direct Covid-19 disability-adjusted life years impact on the Malta population for the first full year. BMC Public Health. 2021;21. 10.1186/s12889-021-11893-4. 10.1186/s12889-021-11893-4 PMC8501913 34627228

