# Supplementary material for: Disability adjusted life years associated with COVID-19 in Denmark in the first year of the pandemic
Source: BMC Public Health. 2022 Jul 9;22:1315. doi: 10.1186/s12889-022-13694-9 (PMC9270752; doi:10.1186/s12889-022-13694-9)
Supplement: Supplementary file 1 — Additional file 1. [file 12889_2022_13694_MOESM1_ESM.pdf]

Disability adjusted life years associated with COVID-19 in Denmark in the first year of the pandemic - Code

R Markdown

This is an R Markdown document. Markdown is a simple formatting syntax for authoring HTML, PDF, and MS Word documents. For more details on using R Markdown see http://rmarkdown.rstudio.com.

When you click the **Knit** button a document will be generated that includes both content as well as the output of any embedded R code chunks within the document. You can embed an R code chunk like this:

```
library(readxl)
library(writexl)

## Warning: package 'writexl' was built under R version 4.0.4

library(tidyverse)

## Warning: package 'tidyverse' was built under R version 4.0.5

## -- Attaching packages --------------------------------------- tidyverse 1.
3.1 --

## v ggplot2 3.3.3     v purrr   0.3.4
## v tibble  3.1.1     v dplyr   1.0.5
## v tidyr   1.1.3     v stringr 1.4.0
## v readr   1.4.0     v forcats 0.5.1

## Warning: package 'ggplot2' was built under R version 4.0.5

## Warning: package 'tibble' was built under R version 4.0.5

## Warning: package 'tidyr' was built under R version 4.0.5

## Warning: package 'readr' was built under R version 4.0.5

## Warning: package 'dplyr' was built under R version 4.0.5

## Warning: package 'forcats' was built under R version 4.0.5

## -- Conflicts ------------------------------------------ tidyverse_conflict
s() --
## x dplyr::filter() masks stats::filter()
## x dplyr::lag()    masks stats::lag()

library(mc2d)

## Loading required package: mvtnorm
```

```
##
## Attaching package: 'mc2d'

## The following objects are masked from 'package:base':
##
##     pmax, pmin

library(data.table)

## Warning: package 'data.table' was built under R version 4.0.5

##
## Attaching package: 'data.table'

## The following objects are masked from 'package:dplyr':
##
##     between, first, last

## The following object is masked from 'package:purrr':
##
##     transpose

library(readr)
library(reshape2)

## Warning: package 'reshape2' was built under R version 4.0.5

##
## Attaching package: 'reshape2'

## The following objects are masked from 'package:data.table':
##
##     dcast, melt

## The following object is masked from 'package:tidyr':
##
##     smiths

library(ggplot2)
library(lubridate)

## Warning: package 'lubridate' was built under R version 4.0.4

##
## Attaching package: 'lubridate'

## The following objects are masked from 'package:data.table':
##
##     hour, isoweek, mday, minute, month, quarter, second, wday, week,
##     yday, year
```

```
## The following objects are masked from 'package:base':
##
##     date, intersect, setdiff, union
```

```r
library(foreach)
```

```
## Warning: package 'foreach' was built under R version 4.0.5
```

```
##
## Attaching package: 'foreach'
```

```
## The following objects are masked from 'package:purrr':
##
##     accumulate, when
```

```r
#Data loading

Positive <- read.csv ("M:/Documents/BoD COVID-19 DK/Data/data_positive_05-11-2021_.csv",sep = ";")

Positive <-as.data.frame(Positive)

#Life expectancy


SEL <- read.csv("M:/Documents/BoD COVID-19 DK/Data/IHME_GBD_2019_TMRLT_Y2021M01D05 (2).CSV")
#Data management
#Dividing cases into age groups (Agegroup)

Positive <- Positive[complete.cases(Positive[ ,"SampleAge"]),]
condition0 <- Positive$SampleAge >= 0 & Positive$SampleAge <=9
condition1 <- Positive$SampleAge >= 10 & Positive$SampleAge <=19
condition2 <- Positive$SampleAge >= 20 & Positive$SampleAge <=29
condition3 <- Positive$SampleAge >= 30 & Positive$SampleAge <=39
condition4 <- Positive$SampleAge >= 40 & Positive$SampleAge <=49
condition5 <- Positive$SampleAge >= 50 & Positive$SampleAge <=59
condition6 <- Positive$SampleAge >= 60 & Positive$SampleAge <=69
condition7 <- Positive$SampleAge >= 70 & Positive$SampleAge <=79
condition8 <- Positive$SampleAge >= 80 & Positive$SampleAge <=89
condition9 <- Positive$SampleAge >= 90


Positive$SampleAge <- Positive$SampleAge

Agegroup <- c()

for (i in 1:nrow(Positive)) {
  if (condition0[i]) {
    Agegroup[i] <- "0-9"
```

```r
  }  else if (condition1[i]) {
    Agegroup[i] <- "10-19"
  }  else if (condition2[i]) {
    Agegroup[i] <- "20-29"
  }  else if (condition3[i]) {
    Agegroup[i] <- "30-39"
  }  else if (condition4[i]) {
    Agegroup[i] <- "40-49"
  }  else if (condition5[i]) {
    Agegroup[i] <- "50-59"
  }  else if (condition6[i]) {
    Agegroup[i] <- "60-69"
  }  else if (condition7[i]) {
    Agegroup[i] <- "70-79"
  }  else if (condition8[i]) {
    Agegroup[i] <- "80-89"
  }  else if (condition9[i]) {
    Agegroup[i] <- "90+"
  }
}

Positive$Agegroup <- Agegroup

remove(condition0)
remove(condition1)
remove(condition2)
remove(condition3)
remove(condition4)
remove(condition5)
remove(condition6)
remove(condition7)
remove(condition8)
remove(condition9)



#Calculating incidence per age group

#Table number of cases per sex and per gender
table(Positive$sex)
```

```
## 
##      F      M 
## 108374 103451
```

```r
table(Positive$Agegroup)
```

```
## 
##   0-9 10-19 20-29 30-39 40-49 50-59 60-69 70-79 80-89   90+ 
## 14235 32422 39802 28981 31067 30639 16797 10485  5601  1794
```

```
#Number of positives by age and gender

Incidence_all <- table(Positive$Agegroup,Positive$sex)
Incidence_all <-as.data.frame.matrix(Incidence_all)

####################

#Hospitalizations (FirstAdmDT)
Positive$hosp <- ifelse((Positive$EpiLPRFirst_inDT != '' ),yes = "1",no = "0"
)
table(Positive$sex,Positive$hosp)

##
##          0      1
##   F 103469   4905
##   M  97565   5886

#Number of hospitalized cases by age and gender (hosp =1)

table(Positive$sex,Positive$Agegroup,Positive$hosp)

## , ,  = 0
##
##
##       0-9 10-19 20-29 30-39 40-49 50-59 60-69 70-79 80-89   90+
##   F  6785 15792 20220 14534 16253 15270  7537  4064  2120   893
##   M  7353 16525 19237 13923 13982 13868  7498  3721  1199   258
##
## , ,  = 1
##
##
##       0-9 10-19 20-29 30-39 40-49 50-59 60-69 70-79 80-89   90+
##   F    54    59   221   299   374   627   678  1098  1119   376
##   M    43    46   124   225   458   874  1084  1602  1163   267

Positive %>% filter(Positive$hosp == "1") %>%
  summarize(table =table(Positive$sex, Positive$Agegroup))

##   table.0-9 table.10-19 table.20-29 table.30-39 table.40-49 table.50-59
## 1      6839       15851       20441       14833       16627       15897
## 2      7396       16571       19361       14148       14440       14742
##   table.60-69 table.70-79 table.80-89 table.90+
## 1        8215        5162        3239      1269
## 2        8582        5323        2362       525

Incidence_hosp <- table(Positive$Agegroup,Positive$sex,Positive$hosp)
Incidence_hosp <-as.data.frame(Incidence_hosp)
Incidence_hosp <- Incidence_hosp %>% filter(Var3 == "1")
Incidence_hosp$Var3 <- NULL
sum(Incidence_hosp$Freq)
```

```
## [1] 10791
```

```
Incidence_hosp_female <- Incidence_hosp %>% filter( Incidence_hosp$Var2 == "F
")
Incidence_hosp_male <- Incidence_hosp %>% filter( Incidence_hosp$Var2 == "M")



Incidence_hosp <- merge(Incidence_hosp_female,Incidence_hosp_male,by="Var1")
remove(Incidence_hosp_female)
remove(Incidence_hosp_male)


#ICU

#Faster version
Positive$ICU <- ifelse((Positive$EpiLprICU_inDT != ''),yes = "1",no = "0")
table(Positive$sex,Positive$ICU)
```

```
##
##          0      1
##   F 107945    429
##   M 102606    845
```

```
#Number of ICU cases by age and gender (ICU =1)

table(Positive$Agegroup,Positive$sex,Positive$ICU )
```

```
## , ,  = 0
##
##
##             F     M
##   0-9    6833  7394
##   10-19 15847 16566
##   20-29 20430 19351
##   30-39 14815 14126
##   40-49 16590 14396
##   50-59 15843 14593
##   60-69  8110  8379
##   70-79  5025  5015
##   80-89  3189  2265
##   90+    1262   520
##
## , ,  = 1
##
##
##             F     M
##   0-9       6     2
##   10-19     4     5
##   20-29    11    10
```

```
##   30-39    18    22
##   40-49    37    44
##   50-59    54   149
##   60-69   105   203
##   70-79   137   308
##   80-89    50    97
##   90+       7     5

Incidence_ICU <- table(Positive$Agegroup,Positive$sex,Positive$ICU)
Incidence_ICU <-as.data.frame(Incidence_ICU)
Incidence_ICU <- Incidence_ICU %>% filter(Var3 == "1")
Incidence_ICU$Var3 <- NULL
sum(Incidence_ICU$Freq)

## [1] 1274

########## Duration####################
covid <- Positive

#ICU duration (years)
covid$ICU_duration <- as.numeric(Positive$Critical)

#### Calculate mean covid$ICU_duration or fit distribution to duration
covid1<-covid %>% filter(covid$ICU=="1"& ICU_duration>"0")
meandurationICU <- mean(covid1$ICU_duration)/365
#The multiplication by 365 is just to interpret results. We need estimates in
years
#In days
summary(covid1$ICU_duration) #in days

##      Min.   1st Qu.    Median      Mean   3rd Qu.      Max.
##   0.00069   4.87960  10.42396  15.36955  20.07292 173.76944

#Hospitalization duration (years)
covid$Severe[is.na(covid$Severe)] <- 0
covid$Critical[is.na(covid$Critical)] <- 0
covid$hosp_duration <- as.numeric(covid$Severe) + as.numeric(covid$Critical)
#### Calculate mean covid$ICU_duration or fit distribution to duration
covid1<-covid %>% filter(covid$hosp=="1"& hosp_duration>"0")
#The multiplication by 365 is just to interpret results. We need estimates in
years

summary(covid1$hosp_duration)

##     Min.  1st Qu.   Median     Mean  3rd Qu.     Max.
##   0.4299   2.1569   5.0722   8.6398   9.7722 287.0000

#Reported in days

#hospital duration excluding people that were moved to ICU
```

```
covid3<- covid1 %>% filter(Critical == "0")
summary(covid3$hosp_duration)

##     Min.  1st Qu.   Median     Mean  3rd Qu.     Max.
##   0.4299   1.9552   4.5472   6.4678   8.0101 287.0000

meandurationhosp_noICU<- mean(covid3$hosp_duration)/365
#Mean duration noICU in years
#############################################################################
######
#### Calculations ######

## simulations
n <- 10^6

set.seed(264)

#Calculating incidence per age group

#Table number of cases per sex and per gender
table(Positive$sex)

##
##      F      M
## 108374 103451

table(Positive$Agegroup)

##
##   0-9 10-19 20-29 30-39 40-49 50-59 60-69 70-79 80-89   90+
## 14235 32422 39802 28981 31067 30639 16797 10485  5601  1794

#Number of positives by age and gender

Incidence_all <- table(Positive$Agegroup,Positive$sex)

Incidence_all <-as.data.frame.matrix(Incidence_all)

write_xlsx(Incidence_all,"Incidence_all.xlsx")


#Proportions table for all cases

prop.table(table(Positive$sex,Positive$Agegroup))

##
##             0-9       10-19       20-29       30-39       40-49       50-5
9
##   F 0.032286390 0.074831345 0.096500380 0.070025446 0.078494781 0.07504850
7
```

```
##   M 0.034915944 0.078230409 0.091401784 0.066791614 0.068170123 0.06959584
2
##
##           60-69       70-79       80-89         90+
##   F 0.038782380 0.024369403 0.015291068 0.005990851
##   M 0.040514958 0.025129471 0.011150819 0.002478484

#We assume the same % of symptomatic for all age groups. ASSUMPTION!

#Proportion of symptomatic cases

sympt <- rbeta(n=369,shape1=216, shape2 = (369-216))

plot(density(sympt))
```

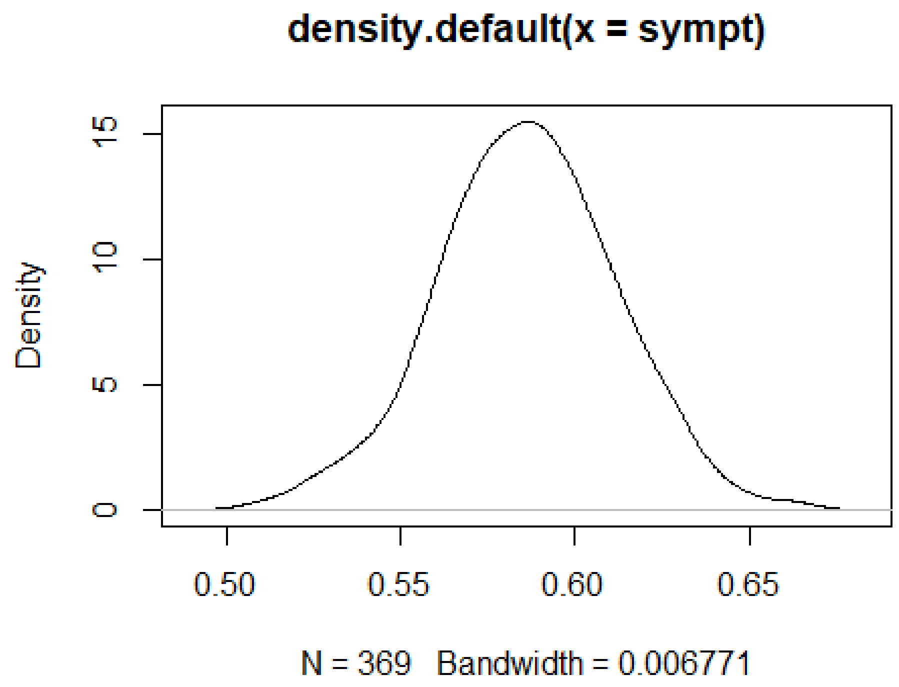


```
#Estimating the number of symptomatic cases per age and sex

#Mean-median-CI
mean_median_ci <-
  function(x) {
    c(mean = mean(x),
      median = median(x),
      quantile(x, probs = c(0.025, 0.975)))
  }

library(foreach)
```

```
Incidence_all$Age <- rownames(Incidence_all)
Incidence_all_melt <- melt(setDT(Incidence_all))

## Using Age as id variables

Incidence_all_symp_mc <- foreach(i = 1:nrow(Incidence_all_melt),.combine = rb
ind) %do% {
  Incidence<- round(Incidence_all_melt$value[i])
  Symptomatic <- sample(x = sympt,size = n,replace = TRUE)
  Incidence * Symptomatic
}

Incidence_all_symp_mc<- cbind.data.frame(Incidence_all_melt$variable,Incidenc
e_all_symp_mc)
Incidence_all_symp_mc<- cbind.data.frame(Incidence_all_melt$Age,Incidence_all
_symp_mc)



rownames(Incidence_all_symp_mc) <- with(Incidence_all_symp_mc, paste(`Inciden
ce_all_melt$Age`,`Incidence_all_melt$variable`,sep = ":"))

summaryinc <- t(apply(X = Incidence_all_symp_mc[,3:ncol(Incidence_all_symp_mc
)],1,FUN = mean_median_ci))

summaryinc <- cbind.data.frame(Incidence_all_symp_mc$`Incidence_all_melt$Age`
,summaryinc)
summaryinc <- cbind.data.frame(Incidence_all_symp_mc$`Incidence_all_melt$vari
able`,summaryinc)



#Incidence_all_symp <- round(Incidence_all*sympt)

#sum(Incidence_all_symp)

write_xlsx(summaryinc,"summaryinc.xlsx")

#################

#Mild/moderate

#duration=10 days, i.e. 0.027 years
#DW=0.051
# pert distribution
#Min= 0.032, Max= 0.074, mode= 0.051
```

```
YLDmildUncertainty <- foreach(i = 1:nrow(summaryinc),.combine = rbind) %do% {
  Mean1 <- summaryinc[i,3]
  DW <- sample(x = rpert(n = 10000, min = 0.032,mode = 0.051,max = 0.074),siz
e = 10.000,replace = TRUE)
  mean_median_ci(Mean1 * DW * 0.027)
}

colSums(YLDmildUncertainty)

##     mean   median     2.5%    97.5%
## 171.5839 169.0345 136.8064 211.0168

#Apply functions to

YLDmild <- summaryinc[,c(3:6)] * 0.027 * 0.051

colSums(YLDmild)

##     mean   median     2.5%    97.5%
## 170.9730 170.9821 155.3005 184.3208

write_xlsx(YLDmild,"YLDmild.xlsx")
####################

#Hospitalizations (FirstAdmDT)

Positive$hosp <- ifelse((Positive$Severe != "0" ),yes = "1",no = "0")

table(Positive$sex,Positive$hosp)

##
##        0    1
##   F   39 4871
##   M   80 5815

#Number of hospitalized cases by age and gender (hosp =1)

table(Positive$sex,Positive$Agegroup,Positive$hosp)

## , ,  = 0
##
##
##      0-9 10-19 20-29 30-39 40-49 50-59 60-69 70-79 80-89  90+
##   F    1     2     1     3     2     1     6    13     9    1
##   M    2     0     1     1     3     8    20    29    15    1
##
## , ,  = 1
##
##
##      0-9 10-19 20-29 30-39 40-49 50-59 60-69 70-79 80-89  90+
```

```
##   F   53    59   221   296   372   626   672  1086  1111  375
##   M   43    46   123   224   456   868  1066  1575  1148  266
```

```
Positive %>% filter(Positive$hosp == "1") %>%
  summarize(table =table(Positive$sex, Positive$Agegroup))
```

```
##   table.0-9 table.10-19 table.20-29 table.30-39 table.40-49 table.50-59
## 1      6839       15851       20441       14833       16627       15897
## 2      7396       16571       19361       14148       14440       14742
##   table.60-69 table.70-79 table.80-89 table.90+
## 1        8215        5162        3239      1269
## 2        8582        5323        2362       525
```

```
Incidence_hosp <- table(Positive$Agegroup,Positive$sex,Positive$hosp)

Incidence_hosp <-as.data.frame(Incidence_hosp)

Incidence_hosp <- Incidence_hosp %>% filter(Var3 == "1")

Incidence_hosp$Var3 <- NULL

sum(Incidence_hosp$Freq)
```

```
## [1] 10686
```

```
Incidence_hosp_female <- Incidence_hosp %>% filter( Incidence_hosp$Var2 == "F
")
Incidence_hosp_male <- Incidence_hosp %>% filter( Incidence_hosp$Var2 == "M")

Incidence_hosp <- rbind.data.frame(Incidence_hosp_female,Incidence_hosp_male)
remove(Incidence_hosp_female)
remove(Incidence_hosp_male)

write_xlsx(Incidence_hosp,"Incidence_hosp.xlsx")

## YLD HOSPITALIZATION

#Duration hosp =meandurationhosp =0.0187

#DW Severe = 0.133

YLDhospUncertainty <- foreach(i = 1:nrow(Incidence_hosp),.combine = rbind) %d
o% {
  Mean1 <- Incidence_hosp[i,3]
  DW <- sample(x = rpert(n = 10000, min = 0.088,mode = 0.133,max = 0.190),siz
e = 10.000,replace = TRUE)
  mean_median_ci(Mean1 * DW * meandurationhosp_noICU)
}

colSums(YLDhospUncertainty)
```

```
##     mean   median     2.5%    97.5% 
## 25.59811 25.11605 20.86523 30.98288

YLDhosp <- t(Incidence_hosp$Freq)  *meandurationhosp_noICU* 0.133

colSums(YLDhosp)

##  [1] 0.1249078 0.1390483 0.5208421 0.6975984 0.8767115 1.4753264 1.5837370
##  [8] 2.5594320 2.6183508 0.8837818 0.1013403 0.1084106 0.2898804 0.5279123
## [15] 1.0746786 2.0456602 2.5122970 3.7118835 2.7055506 0.6268959

sum(YLDhosp)

## [1] 25.18425

# YLDhosp by Sex and Age group


YLDhosp_group <- Incidence_hosp %>% group_by(Var2,Var1) %>%
  summarise(YLDhosp_group = Freq *meandurationhosp_noICU* 0.133)

## `summarise()` has grouped output by 'Var2'. You can override using the `.g
roups` argument.

write_xlsx(YLDhosp_group,"YLDhosp_group.xlsx")

YLDhospUncertainty <- cbind.data.frame(Incidence_hosp[,c("Var1","Var2")],YLDh
ospUncertainty)
write_xlsx(as.data.frame(YLDhospUncertainty),"YLDhospUncertainty.xlsx")

#ICU (ICUAdm)


#Faster version
Positive$ICU <- ifelse((Positive$Critical != "0"),yes = "1",no = "0")

table(Positive$sex,Positive$ICU)

##    
##       1
##   F 429
##   M 845

#Number of ICU cases by age and gender (ICU =1)

table(Positive$Agegroup,Positive$sex,Positive$ICU )

## , ,  = 1
## 
##          
##            F   M
```

```
##   0-9      6   2
##   10-19    4   5
##   20-29  11  10
##   30-39  18  22
##   40-49  37  44
##   50-59  54 149
##   60-69 105 203
##   70-79 137 308
##   80-89  50  97
##   90+     7   5
```

```
Incidence_ICU <- table(Positive$Agegroup,Positive$sex,Positive$ICU)

Incidence_ICU <-as.data.frame(Incidence_ICU)

Incidence_ICU <- Incidence_ICU %>% filter(Var3 == "1")

Incidence_ICU$Var3 <- NULL

sum(Incidence_ICU$Freq)
```

```
## [1] 1274
```

```
write_xlsx(Incidence_ICU,"Incidence_ICU.xlsx")

## YLD ICU

#Duration ICU = meandurationICU = 0.037

#DW Critical = 0.655

YLDICUUncertainty <- foreach(i = 1:nrow(Incidence_hosp),.combine = rbind) %do
% {
  Mean <- Incidence_hosp[i,3]
  DW <- sample(x = rpert(n = 10000, min = 0.579,mode = 0.655,max = 0.727),siz
e = 10.000,replace = TRUE)
  mean_median_ci(Mean * DW * meandurationICU)
}

colSums(YLDICUUncertainty)
```

```
##     mean   median     2.5%    97.5%
## 294.7398 293.4525 279.0179 311.2515
```

```
YLDICU <- t(Incidence_ICU$Freq)  *meandurationICU* 0.655

colSums(YLDICU)
```

```
##  [1] 0.16548584 0.11032389 0.30339071 0.49645752 1.02049602 1.48937257
##  [7] 2.89600222 3.77859338 1.37904868 0.19306681 0.05516195 0.13790487
```

```
## [13] 0.27580974 0.60678142 1.21356284 4.10956506 5.59893763 8.49493985
## [19] 2.67535443 0.13790487

sum(YLDICU)

## [1] 35.13816

# YLDICU by Sex and Age group

#add uncertainty
#0.655 <- rpert(min = 0.569,max = 0.727,n = 10000)

YLDICU_group <- Incidence_ICU %>% group_by(Var2,Var1) %>%
  summarise(YLDICU_group = Freq *meandurationICU* 0.655)

## `summarise()` has grouped output by 'Var2'. You can override using the `.g
roups` argument.

write_xlsx(YLDICU_group,"YLDICU_group.xlsx")



DALY_YLD <- YLDmild + YLDhosp + YLDICU

colSums(DALY_YLD)

##     mean   median     2.5%    97.5%
## 231.2954 231.3045 215.6229 244.6432

YLD_case<- sum(DALY_YLD) /120654

####Uncertainty

DALY_YLD_Uncertainty <- YLDmildUncertainty + YLDhospUncertainty[,3:6] + YLDIC
UUncertainty
DALY_YLD_Uncertainty <- cbind.data.frame(YLDhospUncertainty[,1:2],DALY_YLD_Un
certainty)
colSums(DALY_YLD_Uncertainty[,3:6])

##     mean   median     2.5%    97.5%
## 491.9218 487.6031 436.6895 553.2512

##################### DEATHS #####################

#sub-set of data with patients that died

#Calculations with ALL deaths, with variable used to extract data "date of de
ath"
deaths <- Positive %>% filter(as.numeric(Positive$death30days_final) != "NA")

plot(deaths$SampleAge,as.factor(deaths$Agegroup))
```

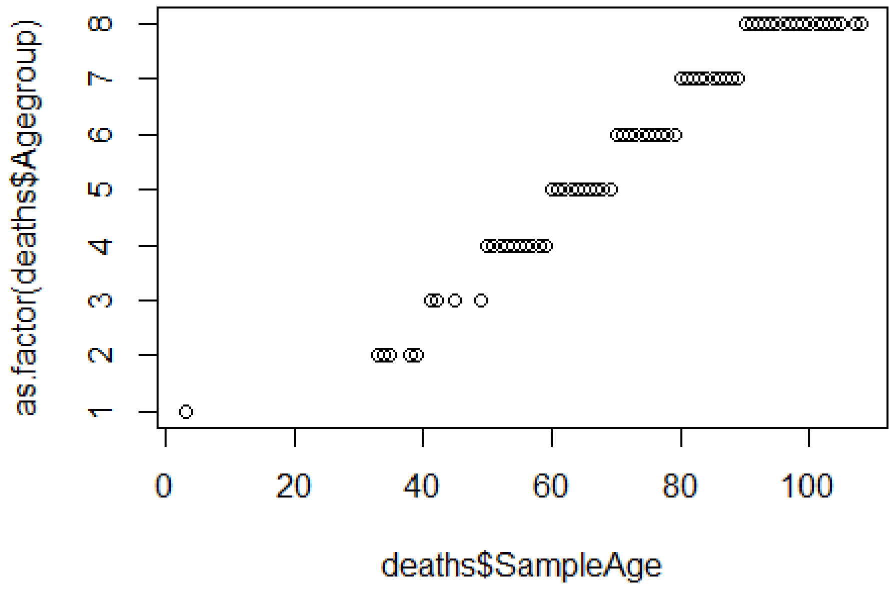


```
#Age of patients =SampleAge

#Number of deaths per age group

numb_deaths <- table(deaths$casedefinition,deaths$SampleAge,deaths$sex)

sum(numb_deaths)

## [1] 2383

table(deaths$sex,deaths$Agegroup)

##
##      0-9 30-39 40-49 50-59 60-69 70-79 80-89 90+
##    F   0     4     2    21    73   228   439 323
##    M   1     2     4    32   122   394   518 220

lifeexpect_mod <- lm (SEL$Life.Expectancy~SEL$Age)

lifeexpect_byyear <- seq(1,107,by=1)

#calculating remaining LE

RLE <- as.data.frame(sapply(lifeexpect_byyear, function(x) {
  (x *(-0.9163)) + 87.217
} ))
colnames(RLE)<-"RLE"
```

```
RLE$RLE[RLE$RLE <0] <-0.1685


#plot(lifeexpect_mod)


#Assumption: we ingnored if a person celebrated their birthday at the hospita
l (before dying)

#multiply freq of deaths by age by the corresponding RLE

RLE$SampleAge <-lifeexpect_byyear

colnames(RLE) <- c("RLE","SampleAge")

#THIS IS NOT WORKING - RLE DOESNT MATCH AGE GROUP
#WE HAVE TO MATCH THE AGE OF DEATH WITH RLE

YLL_person <- merge(deaths,RLE,by="SampleAge")

plot(YLL_person$SampleAge,YLL_person$RLE)
```

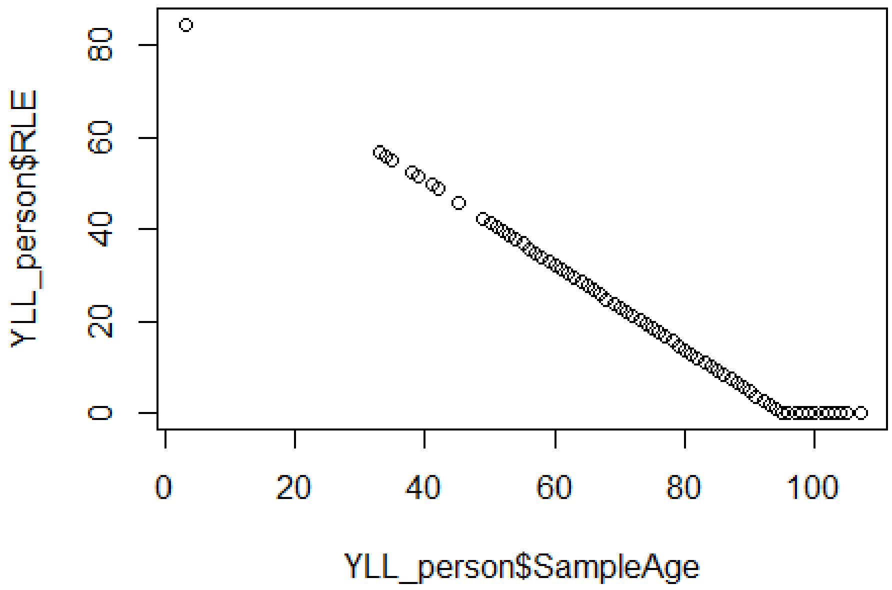


```
YLL_person$Agegroup <-as.factor(YLL_person$Agegroup)

plot(YLL_person$SampleAge,YLL_person$Agegroup)
```

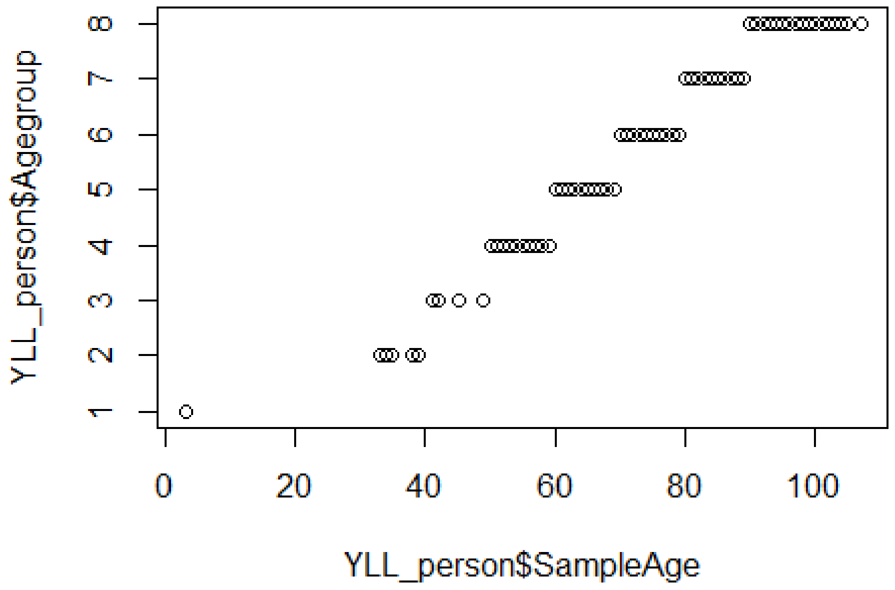


```
Yll_peragegroup <-aggregate(YLL_person$RLE~YLL_person$Agegroup,FUN = sum)

totalYLL <-sum(YLL_person$RLE)

#YLL per Age and Sex

YLL_group <- YLL_person %>% group_by(sex,Agegroup) %>%
  summarise(YLL_group =sum(RLE))

## `summarise()` has grouped output by 'sex'. You can override using the `.gr
oups` argument.

YLL_group <- merge.data.frame(DALY_YLD_Uncertainty[,1:2],YLL_group,
                              by.x = c("Var1","Var2"),by.y = c("Agegroup","se
x"),all = TRUE)
YLL_group[is.na(YLL_group)] <- 0

YLL_group <- YLL_group[ order(YLL_group$Var2), ]

write_xlsx(YLL_group,"YLL_group.xlsx")

#Danish pop =5806081

#Total DALY

DALY <-(colSums(DALY_YLD) + totalYLL)
```

```
#Total DALY

DALY_GROUP <- cbind.data.frame(DALY_YLD_Uncertainty[,1:2],
                               apply(X = DALY_YLD_Uncertainty[,3:6], MARGIN =
2,FUN = function(i) i + YLL_group$YLL_group))

colnames(DALY_GROUP) <- c("Age","Sex","mean","median","2.5%","97.5%" )

write_xlsx(DALY_GROUP,"DALY_GROUP.xlsx")

#Total DALY per 100,000

DALY_100 <-(sum(DALY_YLD) + totalYLL)/58
DALY_100

## [1] 527.7919
```

Note that the `echo = FALSE` parameter was added to the code chunk to prevent printing of the R code that generated the plot.
