# Supplementary material for: Disability adjusted life years associated with COVID-19 in Denmark in the first year of the pandemic
Source: BMC Public Health. 2022 Jul 9;22:1315. doi: 10.1186/s12889-022-13694-9 (PMC9270752; doi:10.1186/s12889-022-13694-9)
Supplement: Supplementary file 2 — Additional file 2: Table S1. Years of life lost due to disability (YLD) caused by mild, severe and critical symptoms by age and sex of COVID-19 in Denmark, February 2020 to February 2021 (Mean and 95% Confidence Interval). [file 12889_2022_13694_MOESM2_ESM.docx]

**Disability adjusted life years associated with COVID-19 in Denmark in the first year of the pandemic**

**Additional File 2**

Table S1. Years of life lost due to disability (YLD) caused by mild, severe and critical symptoms by age and sex of COVID-19 in Denmark, February 2020 to February 2021 (Mean and 95% Confidence Interval).

|  | YLD Mild | | | | | | YLD Severe | | | | |  | YLD Critical | | | | |  |
| --- | --- | --- | --- | --- | --- | --- | --- | --- | --- | --- | --- | --- | --- | --- | --- | --- | --- | --- |
| **Age** | **Female** | | | **Male** | | | **Female** | | | **Male** | | | **Female** | | | **Male** | | |
|  | **Mean** | **95% CI** | | **Mean** | **95% CI** | | **Mean** | **95% CI** | | **Mean** | **95% CI** | | **Mean** | **95% CI** | | **Mean** | **95% CI** | |
| **0-9** | 5.5 | 5.0 | 6.0 | 6.0 | 5.4 | 6.4 | 0.1 | 0.1 | 0.1 | 0.1 | 0.1 | 0.1 | 1.5 | 1.4 | 1.6 | 1.2 | 1.1 | 1.3 |
| **10-19** | 12.8 | 11.6 | 13.8 | 13.4 | 12.1 | 14.4 | 0.1 | 0.1 | 0.2 | 0.1 | 0.1 | 0.1 | 1.6 | 1.6 | 1.7 | 1.3 | 1.2 | 1.4 |
| **20-29** | 16.5 | 15.0 | 17.8 | 15.6 | 14.2 | 16.8 | 0.5 | 0.4 | 0.6 | 0.3 | 0.2 | 0.3 | 6.0 | 5.7 | 6.3 | 3.3 | 3.1 | 3.6 |
| **30-39** | 12.0 | 10.9 | 12.9 | 11.4 | 10.4 | 12.3 | 0.7 | 0.6 | 0.8 | 0.5 | 0.4 | 0.7 | 8.0 | 7.4 | 8.4 | 6.2 | 5.8 | 6.5 |
| **40-49** | 13.4 | 12.2 | 14.5 | 11.7 | 10.6 | 12.6 | 0.9 | 0.8 | 1.1 | 1.0 | 0.9 | 1.2 | 9.9 | 9.5 | 10.5 | 12.9 | 12.2 | 13.8 |
| **50-59** | 12.8 | 11.7 | 13.8 | 11.9 | 10.8 | 12.8 | 1.5 | 1.3 | 1.8 | 2.1 | 1.7 | 2.5 | 17.5 | 16.3 | 18.2 | 23.6 | 22.3 | 24.5 |
| **60-69** | 6.6 | 6.0 | 7.1 | 6.9 | 6.3 | 7.5 | 1.7 | 1.4 | 2.1 | 2.7 | 2.1 | 3.2 | 18.7 | 18.0 | 19.4 | 29.5 | 28.2 | 30.8 |
| **70-79** | 4.2 | 3.8 | 4.5 | 4.3 | 3.9 | 4.6 | 2.6 | 2.2 | 3.3 | 3.8 | 3.1 | 4.6 | 30.0 | 28.9 | 32.2 | 43.1 | 39.9 | 46.1 |
| **80-89** | 2.6 | 2.4 | 2.8 | 1.9 | 1.7 | 2.1 | 2.6 | 2.2 | 3.0 | 2.6 | 2.1 | 3.2 | 30.9 | 29.5 | 32.8 | 32.1 | 30.9 | 33.4 |
| **90+** | 1.0 | 0.9 | 1.1 | 0.4 | 0.4 | 0.5 | 0.9 | 0.7 | 1.1 | 0.6 | 0.5 | 0.8 | 10.3 | 9.4 | 10.9 | 7.4 | 6.8 | 8.0 |
| **Total** | 87.5 | 79.5 | 94.3 | 83.5 | 75.8 | 90.0 | 11.8 | 9.7 | 14.2 | 13.8 | 11.2 | 16.7 | 134.3 | 127.5 | 141.9 | 160.4 | 151.5 | 169.3 |
